# Experiments Testing the Causes of Namibian Fairy Circles

**DOI:** 10.1371/journal.pone.0140099

**Published:** 2015-10-28

**Authors:** Walter R. Tschinkel

**Affiliations:** Department of Biological Science, Florida State University, Tallahassee, Florida, United States of America; NERC Centre for Ecology & Hydrology, UNITED KINGDOM

## Abstract

The grasslands on the sandy soils of the eastern edge of the Namib Desert of Namibia are strikingly punctuated by millions of mostly regularly-spaced circular bare spots 2 to 10 m or more in diameter, generally with a margin of taller grasses. The causes of these so called fairy circles are unknown, but several hypotheses have been advanced. In October 2009, we set up experiments that specifically tested four hypothesized causes, and monitored these 5 times between 2009 and 2015. Grass exclusion in circles due to seepage of subterranean vapors or gases was tested by burying an impermeable barrier beneath fairy circles, but seedling density and growth did not differ from barrier-less controls. Plant germination and growth inhibition by allelochemicals or nutrient deficiencies in fairy circle soils were tested by transferring fairy circle soil to artificially cleared circles in the grassy matrix, and matrix soil to fairy circles (along with circle to circle and matrix to matrix controls). None of the transfers changed the seedling density and growth from the control reference conditions. Limitation of plant growth due to micronutrient depletion within fairy circles was tested by supplementing circles with a micronutrient mixture, but did not result in differences in plant seedling density and growth. Short-range vegetation competitive feedbacks were tested by creating artificially-cleared circles of 2 or 4 m diameter located 2 or 6 m from a natural fairy circle. The natural circles remained bare and the artificial circles revegetated. These four experiments provided evidence that fairy circles were not caused by subterranean vapors, that fairy circle soil *per se* did not inhibit plant growth, and that the circles were not caused by micronutrient deficiency. There was also no evidence that vegetative feedbacks affected fairy circles on a 2 to 10 m scale. Landscape-scale vegetative self-organization is discussed as a more likely cause of fairy circles.

## Introduction

The eastern edge of the Namib Desert (the Pro-Namib) is home to a sparse grassland of *Stipagrostis obtusa* and *S*. *uniplumis* punctuated by millions of quasi-circular, mostly regularly-spaced bare areas from 2 to 12 or more meters in diameter, often somewhat concave, and with a perimeter of taller grass (usually *S*. *ciliata* and *S*. *giessii*). These so-called "fairy circles" are clearly visible on Google Earth and occur from southern Angola to northern South Africa in a narrow band in which the rainfall is between 50 and 150 mm per year and the soils are sandy. Within their range, the average size of circles decreases with increasing latitude [1–4]. Since the early 1970s, many hypotheses for the formation of these mysterious vegetation gaps have been proposed. These included feeding by termites(including the sand termite, *Psammotermes allocerus*), subterranean volatile or toxic substances, soil radioactivity, nutrient deficiencies, inhibitory soil properties and plant competitive feedbacks including those leading to vegetative self-organization. In 2004, van Rooyen et al. [1] (whose review should be consulted for earlier literature) evaluated all hypotheses that had been published at the time, and found that almost none were supported by the weight of available evidence. Most of the accumulated evidence is correlative in nature, depending on the co-occurrence of a biotic or physical factor and the fairy circles. Several proposed causes of fairy circles post-date the van Rooyen et al. review [1]. These include ants [5], but the putative species are homopteran-tenders whose range is far greater than the range of fairy circles. Geochemical hydrocarbon seeps (along with unresolved other compounds) or toxic subterranean volatiles were proposed by Naudé et al. [6] and Jankowitz et al. [7], respectively. Neither of these more recent hypotheses seem likely to produce the large barren patches and regular dispersion of fairy circles, nor have hydrocarbons been shown to influence plant growth, except at very high concentrations [8, 9].

The bare circles usually have a higher soil water content than the vegetated matrix, presumably because there are no plants to deplete it [2, 4, 10]. The nutritional differences between fairy circle and matrix soils are less clear. Across different sites, van Rooyen et al. [1] and Moll [3] failed to detect consistent differences between fairy circle and matrix soils. In contrast, by sampling across a relatively homogeneous area, Cramer and Barger [2] found small but significant differences in soil macro-nutrients between the fairy circles and the matrix. Considering the small differences, these authors concluded that they were likely a consequence of the long-term persistence of the vegetation pattern, rather than a cause of it.

Tests of inhibition of plant growth by fairy circle soil have produced mixed results, with some authors reporting inhibition, and others not [3, 10, 11], while still others found mixed outcomes, depending on when the soil was collected, and its source [1]. These tests were carried out either *in situ* by burying pots with either open or closed bottoms in the soil in fairy circles and the matrix [7], or in pots in glass houses [1]. Reduced growth of plants in open pots in circles was concluded to result from a semi-volatile gas [7], although no attempt was made to break the soil water connection between those pots and the surrounding soil. In glass house experiments, phytometers (i.e. plants not native to the region where soils were collected) were generally found to accumulate less biomass on soils collected from the fairy circles than those collected from the matrix [1, 2]. Phytometers grew best on soils collected from around *Euphorbia damarana*, indicating that the reduced growth on fairy circle soils was unlikely to be the consequence of allelopathic chemicals released by this species [1]. Cramer and Barger [2] concluded that the reason for reduced growth of phytometers in soils from the fairy circles relative to those from the matrix was likely smaller concentrations of C and N in those soils, although they did not measure or consider micro-nutrients as a potential cause.

Although van Rooyen et al. [1] judged termites to be an unlikely causal agent for a range of reasons, Juergens [4, 12] recently asserted that fairy circles were caused by the sand termite, *Psammotermes allocerus*. His conclusion was based only on correlations of fairy circles with signs of termite activity, but without experimental manipulations. Juergens [12] recently suggested that production of methane by the termites might play a role in fairy circle formation and maintenance, but the concentrations he reported are 1/2000th to 1/50,000th of the concentrations reported by Smith et al. [9] to produce chlorosis or growth reduction in plants. Vlieghe et al. [13] offered a causal mechanism whereby the termites killed the grass by eating the roots, but their experiments were carried out with wheat seedlings under starvation conditions. Moreover, Zeidler [14] classified *Psammotermes* as a wood and litter feeder, a conclusion also supported by Crawford and Seeley [15]. These studies thus failed to provide either a biological mechanism for the effects of termites or an adaptive reason for the maintenance of large barren patches by the termites. Along this line, Tschinkel [16] found no association between fairy circles and the endemic Namibian termite, *Baucaliotermes hainesi*, as would have been expected if this species were involved in fairy circle formation or maintenance.

Fairy circles are not permanent features as was first noted by Albrecht et al. [10]. Tschinkel [17] estimated circle lifespans of about 40 years on the basis of turnover (births and deaths) over a span of 4 years. Juergens ([4], supplementary online material) suggested a much longer lifespan, but the dynamics of his site were rather different, with new circles greatly exceeding vanished circles. Vlieghe et al. [13] described the appearance and growth of fairy circles on an annual scale. The dynamic nature of the fairy circles makes it unlikely that they are the product of a static or rapidly fluctuating pattern-forming mechanism, such as a geochemical source of volatiles [6].

Cramer and Barger [2] determined the local conditions of rainfall, seasonal variation and soil associated with fairy circles at several sites, and showed that these factors successfully predicted the occurrence of fairy circles on a regional scale, identifying a narrow band where these conditions pertained. Moreover, they showed that the mean size of fairy circles increased with aridity, a finding in keeping with predictions based on models employing facilitation-competition interactions [18, 19]. This suggests that fairy circles are a climate-dependent emergent phenomenon, especially considering that the narrow range is only a tiny fraction of the total range of the grass species involved. The strong link to climate and the regional variability in climate may partially explain the dynamic nature of the fairy circles. Moreover, they showed that these physical conditions also predict the mean size, spacing and landscape coverage of the circles, and that the circles are overdispersed (i.e. form a regular, hexagonal pattern) only at high landscape occupancy rates, leading to the conclusion that fairy circles may be the product of the self-organization.

Evidence for self-organization through some kind of activation-inhibition feedback has been growing, with several mathematical models producing congruence with spatial analyses and suggesting possible mechanisms. Models in which plants draw some limiting resource to themselves thereby impoverishing more distant soil create a short-range positive feedback (i.e. facilitation) and a long-range negative (i.e. competitive) feedback. Such self-organization models have been shown to result in a variety of vegetation spatial patterns whose specific geometry (bands, spots, stripes, gaps) depend on the relative scale of the positive and negative feedbacks [18–28]. Lejeune and Tlidi [20, 21] published the first models that predicted gaps like those of fairy circles.

The regular patterning of fairy circles has been commonly measured. However, aside from noting their overdispersion, there have been few analyses of the rich details of these spatial patterns. Spatial analysis of aerial images by Getzin et al. [28] confirmed that fairy circles were strongly overdispersed, and that the quantitative details of these patterns were remarkably homogeneous over large scales and geographic locations. Furthermore, the pattern analysis revealed high-order effects in which the regular patterning propagates from one neighbor to the next. Fairy circle size was negatively correlated up to 13 m away, suggesting neighborhood interaction among circles. A partial-differential equation model based on plant competition for resources (mostly water) accounted quantitatively for the major features of the observed fairy circle patterns. These findings support the hypothesis that fairy circles are an example of self-organization through the feedbacks created by competition for scarce resources, probably primarily the water that limits productivity in this arid land. More recently, Zelnik et al. [29] showed that models could also account for temporal variation in fairy circle birth, death and size. These results emphasize that any proposed causal process needs to account for all of the attributes of fairy circles, including their regional distribution, variations in size, landscape occupancy, dispersion and life cycles. Most of the proposed causes cannot account for most of these properties (see Discussion).

Here we report the results of five-year experiments established in 2009 to test four different hypotheses of fairy circle formation. These were (1) subterranean toxic vapors or gases; (2) micronutrient depletion; (3) plant inhibition by fairy circle soil; (4) fairy circle neighborhood interactions.

## Materials and Methods

### Experimental sites

#### Ethical Statement

The individual shown in figures has given written informed consent (as outlined in PLOS consent form) for use of these images. The work was carried out under permits from the NamibRand Nature Reserve. No protected species were involved.

All experiments were sited and carried out at the privately owned NamibRand Nature Reserve (NRNR) in the pro-Namib Desert of west-central Namibia, under Namibian Ministry of Environment and Tourism Permit No. 1422/2009. The NRNR is topographically diverse, including rocky mountains, alluvial deposits, gravel plains, mobile aeolian dunes and sand plains of red Kalahari sand. Fairy circles occur only on the last (although a few circles on stony substrate or gravel plains have been reported [30]. Vegetation on these sandy plains is dominated by three species of grasses, *Stipagrostis ciliata*, *S*. *obtusa*, and *S*. *uniplumis*, but several species of dicoyledons were also present. The vegetation is dry and brown except for several weeks after infrequent adequate rains. Rain is brought to the region mostly by a summer monsoon, averaging 70 to 80 mm/yr, but this varies greatly from year to year. Mean annual temperature is about 22°C with monthly means ranging from 15 to 16°C in winter to 26 to 27°C in summer. Several large grazers inhabit the area, including oryx antelope, zebra, springbok, ostrich, hartebeest and giraffe (which do not occur on the sandy plains). Animal tracks and evidence of grazing are abundant.

**Fig 1:**
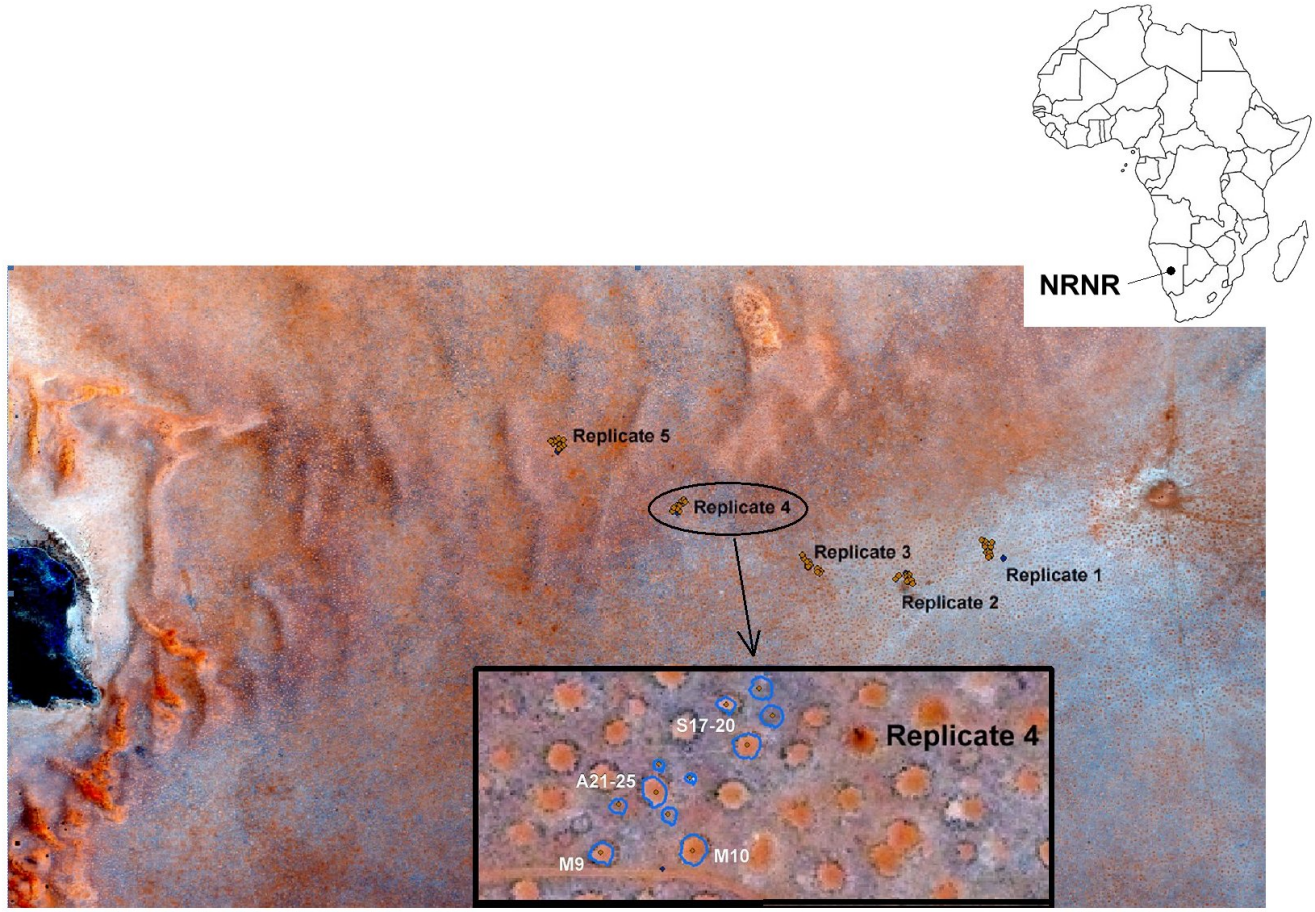
Location of the five replicate sets of experimental manipulations, showing the labeled detail of one of the replicate sets. The barrier treatments and controls were located only at Replicates 1 and 2. S = soil transfer experiment; A = artificial neighborhood experiment; M = micronutrient experiment. The experimental area is an undulating sandy plain sloping to the east, with scattered small dunes. Reprinted and modified from satellite image Namibia WV3 08DEC15 1010010008EC6A08 under a CCBY license, with permission from Digital Globe, Inc., original copyright 2008.

Four experiments each with five replicates were set up along a track that traversed a rolling sandy plain that rose gradually over 6 km from a pan at 960 m elevation in the east to the margin of a rocky outcrop (Jagkop) at 1100 m elevation in the west. Fig 1 shows the locations of the 5 replicate sets, each of which contains one replicate of each of the 4 experiments (except for the Barrier Experiment, of which there are only 2 replicates. Rain gauges have been set up and monitored by the reserve staff. Locations and treatments of all plots can be found in S1 Table.

### Experimental descriptions

#### Barrier Experiment

**Fig 2:**
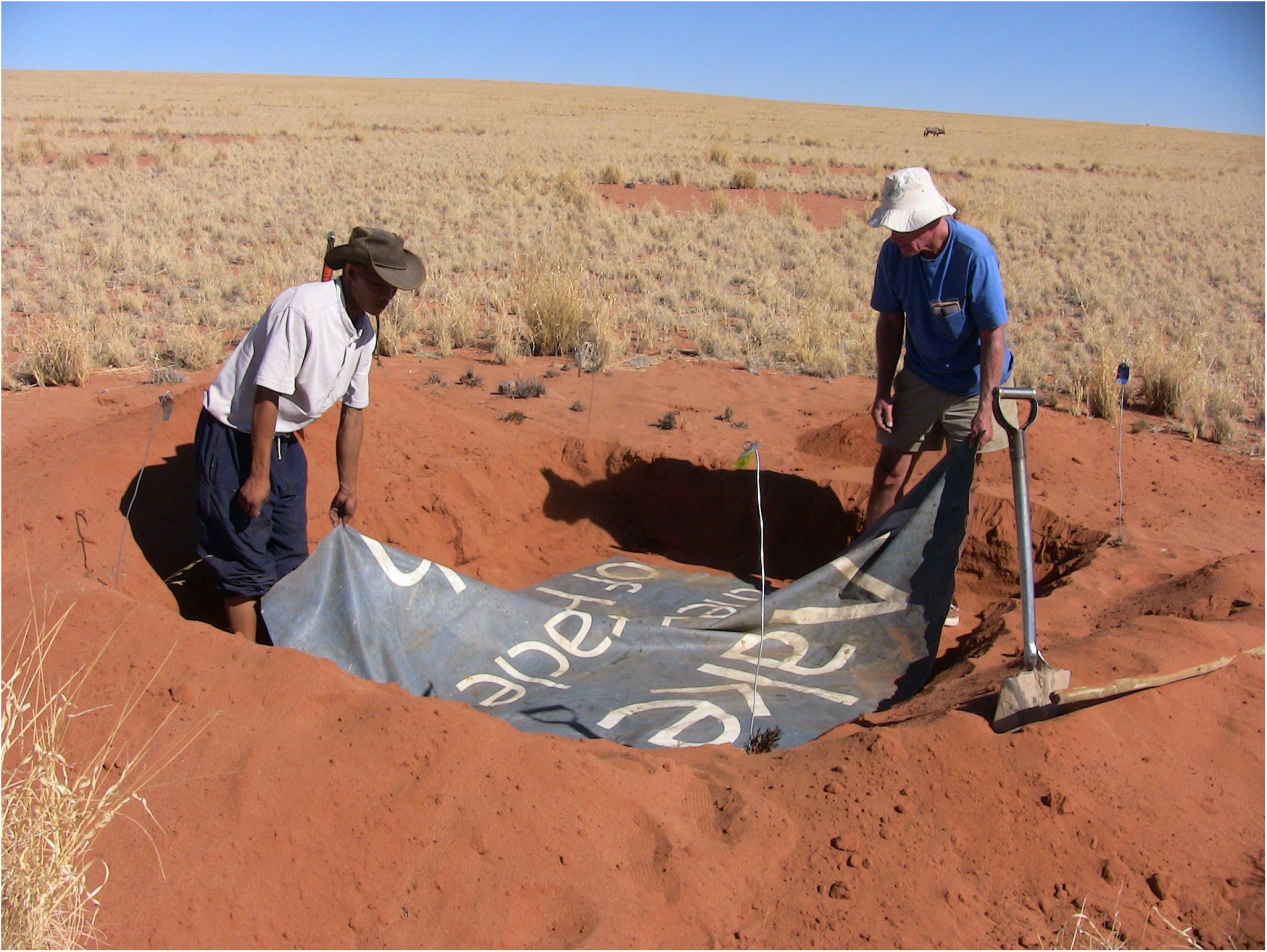
Set-up of the barrier experiment. The top 30 cm of soil was removed, a heavily rubberized tarp (donated by the NamibRand Nature Reserve) laid in the bottom and the soil replaced. Controls were similarly excavated but the soil was replaced without installing a tarp. Person in blue T-shirt is the author. Informed consent has been obtained from the other person.

This experiment was a test of the hypothesis [6, 7, 10] that toxic vapors or hydrocarbon gases emanate from deep in the soil of fairy circles, and that these vapors inhibit plant germination, seedling density and growth. We blocked or reduced vapor movement upward (and coincidentally, water movement downward) by digging out a section of circle soil 2.5 m square to a depth of 30 cm, placing a heavily rubberized tarp, more or less impermeable to water and gases, and returning the excavated soil on top of the sheet and leveling it (Fig 2). The controls were simply excavated to a similar depth, and the soil returned and leveled. Only two replicates were set up, one each at Replicate Sites 1 and 2 in Fig 1 (coordinates and treatments in S1 Data).

#### Micronutrient Experiment

Macronutrient differences between circle and matrix soil have been inconsistent and small, but micronutrients have rarely been tested. The rationale for this experimental design was that if circles were bare because the soil was depleted in micronutrients, then adding these micronutrients should result in vegetation growth within the circle. Moreover, micronutrients are easily applied and tested. The components of the micronutrient mixture (Table 1) were obtained separately and dissolved together in water to make a stock solution at 10-fold the final concentration. Relative amounts of each were approximated from the literature. For application, this stock was diluted 10-fold and sprinkled on the treatment circles at the rate of 1 L per m^2^, consequently depositing 2.3 g of the mixture per m^2^. At this irrigation rate the wetting front extended to 10 cm depth resulting in a total micronutrient addition of 11 mg kg^-1^ of soil (assuming a bulk density of about 1.5). Rates for individual micronutrients are simply their proportion of the total mixture.

**Table 1:** Composition of the micronutrient mixture. Values were determined from approximate ranges occurring in a variety of natural and agricultural systems.

element	chemical compound	g compound per kg mixture	Element (mg L^-1^ soil)
Co	Cobalt dichloride dihydrate	13	55
Cu	Cupric(II) sulfate pentahydrate	16	60
Fe	Ferric chloride	193	733
Mn	Manganese sulfate 2 H_2_O	381	1341
Mo	Ammonium molybdate	5	31
Zn	Zinc chloride	44	233
B	Boric acid	294	567
Cr	Chromium chloride	51	185

Initially, five pairs of neighboring circles were chosen for each replicate, one pair at each replicate site on the Jagkop track (Fig 1 and coordinates in S1 Data). Four additional replicates were set up on the sandy plain *ca*. 16 km farther north. One circle of each replicate pair was designated the treatment and the other the control. The treatment received the diluted micronutrient solution, and the control received only water. Because the wind probably reduces the seed bank in the bare circles, seeds were raked together from the nearby matrix, spread over both circles of the pairs, and raked in to reduce wind drift.

#### Soil transfer experiment

**Fig 3:**
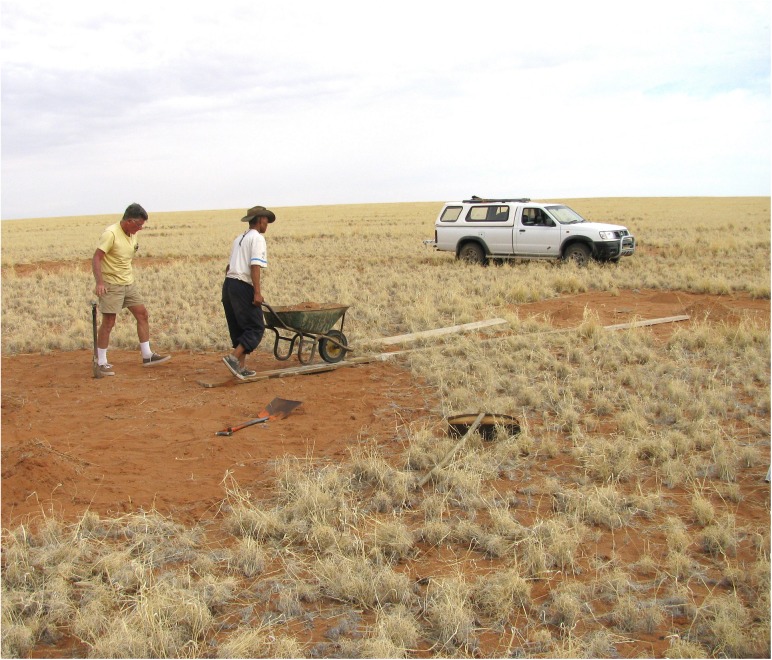
Transferring soil from an artificial circle (left) to a natural circle (right). The top 10 cm of soil was transferred according to the scheme in Fig 4 and leveled. Artificial circles were created by uprooting and removing grass. Five replicate sets were completed. Person in yellow T-shirt is the author. Informed consent has been obtained from the other person.

**Fig 4:**
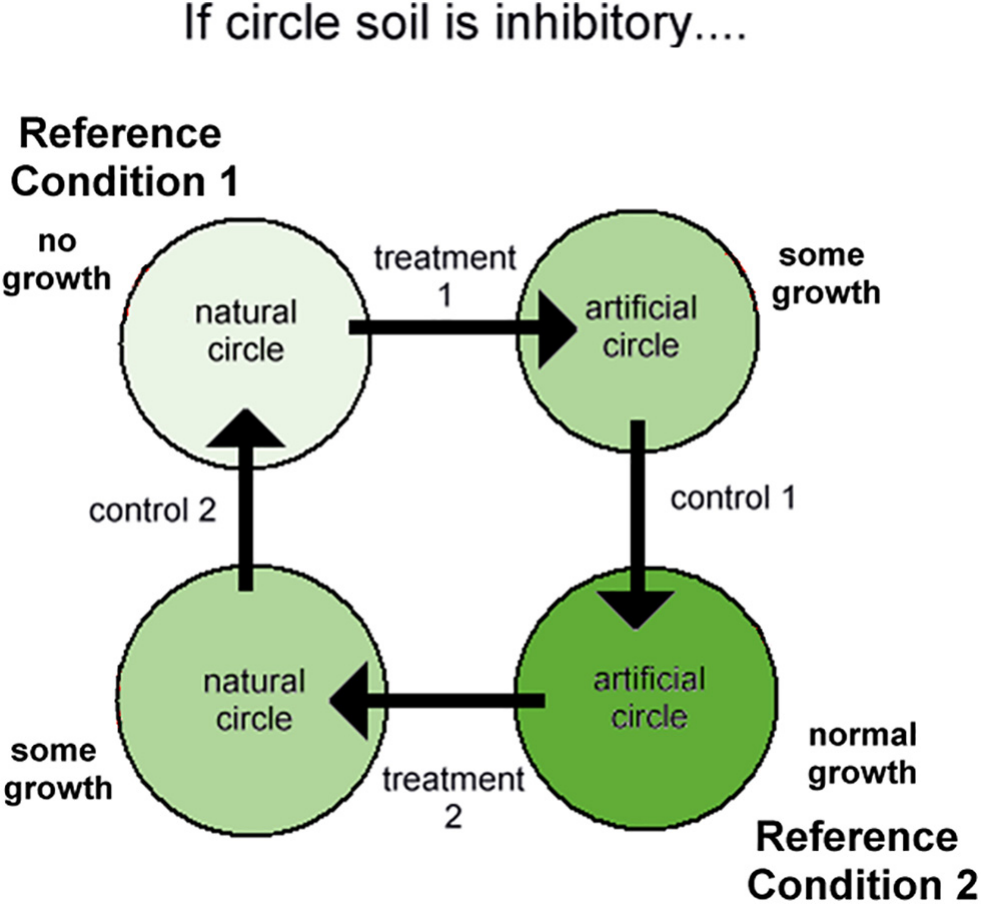
The rationale and procedure of the soil transfer experiment. If circle soil is inhibitory, the two control reference conditions (circle to circle transfer; matrix to matrix transfer) should see little change from the original circle and matrix conditions, respectively, but circle to matrix transfer should inhibit matrix growth, and matrix to circle transfer should disinhibit circle growth.

The rationale for this experimental design was that if fairy circle soil inhibited plant germination or growth through either nutritional or allelochemical mechanisms, then transfer of this soil to the matrix should reduce plant growth, while matrix soil transferred to fairy circles should stimulate growth (Fig 3). Controls (circle to circle; matrix to matrix) should result in little change. Each of the five replicate sets consisted of two adjacent natural circles and two circles of similar size cleared of grass in the nearby matrix (artificial circles). Surface sand to a depth of about 10 cm in each circle was raked into piles, and the piles transferred as follows: matrix (artificial circle) to natural circle; natural to natural; natural to matrix; matrix to matrix. This produced 2 treatments (matrix to natural; natural to matrix) and 2 controls (matrix to matrix; natural to natural) (Fig 4). The transferred soil was spread out evenly in the circles (coordinates in S1 Data).

#### Neighborhood test with artificial circles

**Fig 5:**
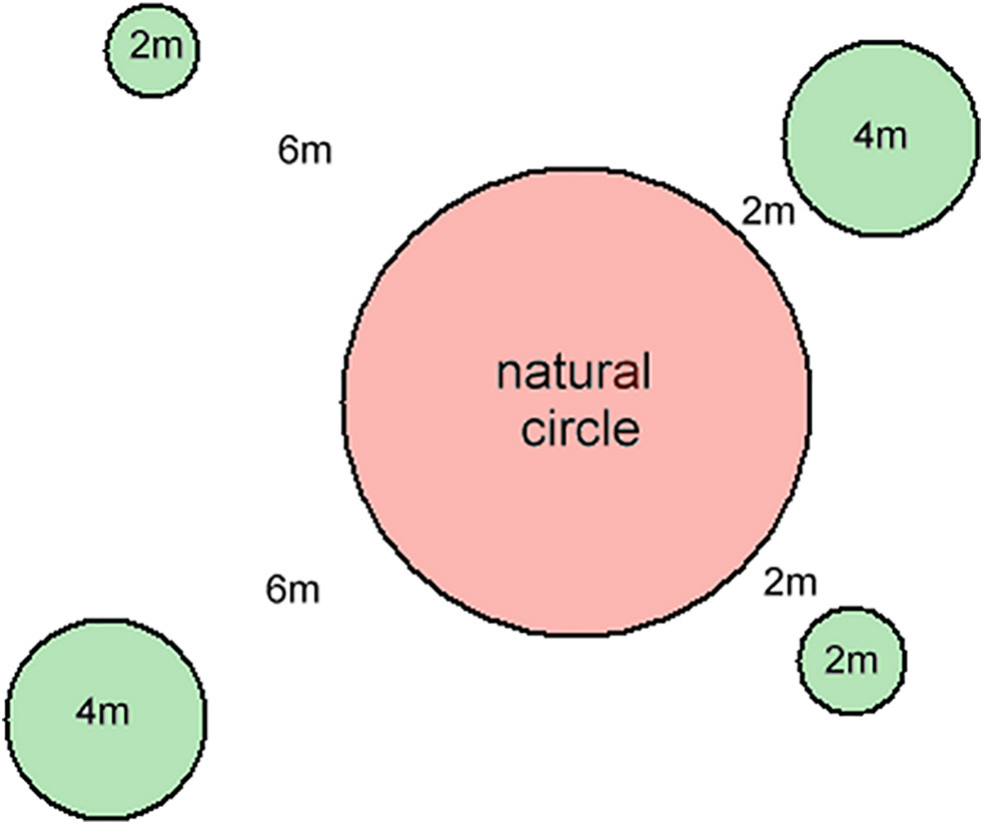
Layout of the neighborhood experiment with artificial circles of two sizes and two distances from a natural circle. If circles interact in neighborhoods on this scale, the fates of the artificial circles should depend on their distance from the natural circle and their initial diameter.

The rationale for this experimental design was that if circles interact, plant growth in the artificial circles should vary with distance from the existing circles, and possibly with size. Natural fairy circles were chosen, and artificial circles of two sizes (2 m, 4 m diameter) were created at two distances from the existing circle (2 m, 6 m) by uprooting the grass (Fig 5, coordinates in S1 Data). The diameters of the central fairy circles were (in order, from replicate 1 to 5): 7.5 m; 9.5 m; 7.5 m; 7.5 m; 7.2 m.

### Monitoring

Each circle was visited in February and May of 2010 and 2011 for vegetation monitoring and photography, with a final inspection and photography visit in February 2015. Given sufficient rain, these checks covered the period from germination to the end of the growing season. Dependent measures included (1) seedling density per m^2^ or per circle; (2) grass/dicotyledon mixture; (3) mean height of grass within circle and circle perimeter; (4) visual estimate of general health of the plants in circles on a 4-point scale from poor/dying/dead to green/luxuriant. Perimeter grass health was judged on a similar scale, with seed production added as a condition for very good health. When seedling density was low, total counts of circles were made, when they were high, several haphazard m^2^ samples were counted and averaged. All circles were photographed as stereo pairs before sampling, forming a visual record of circle condition. Monitoring data are provided in S1 Data.

### Data analysis

Germination data were expressed as seedlings per m^2^ for each check date and analyzed as a repeated measure analysis of variance (ANOVA) by treatment for each experiment using Statistica 64 (http://software.dell.com/products/statistica/). Plant height was similarly analyzed. An index of biomass was computed as the product of seedling density and height.

## Results

In the arid environment of the NamibRand Nature Reserve, all plant growth closely follows rainfall. In 2010, the first summer after the establishment of the experiments, the rain gauge closest to the experimental replicates (1 to 3 km) indicated only a modest rainfall, with a total of 78 mm from January to May (Jan 28 mm; Feb 28 mm; Mar 8 mm; Apr 0 mm; May 8 mm). Although this was enough to stimulate germination and some growth, it did not sustain it for long. In contrast, the summer of 2011 was a season of exceptional rainfall, with the same gauge collecting a total of 370 mm, almost 5 times as much as the previous year falling in the experimental area from January to May (Jan 56 mm; Feb 122 mm, Mar 107 mm; Apr 6 mm; May 80 mm). This abundant rain produced exuberant plant growth, and activated the experimental treatments. Vegetation monitoring confirmed this seasonal and inter-annual variation in plant growth. Mean seedling density of grasses at the 59 monitored sites in February of 2010 was 3.34 per m^2^, while in February of 2011 it was 17-fold higher (57 per m^2^). Herb seedling density rates were 0.35 and 39 per m^2^, respectively in 2010 and 2011. Plant height (excluding perimeter grass) averaged 6.84 cm in May of 2010, while in May of 2011 it averaged 24 cm. Perimeter grasses were about 4 times as tall in May 2011 than in February 2010 (n = 37 sites). Perimeter grasses grew 10-fold in height between February and May 2011.

### Barrier experiment

**Fig 6:**
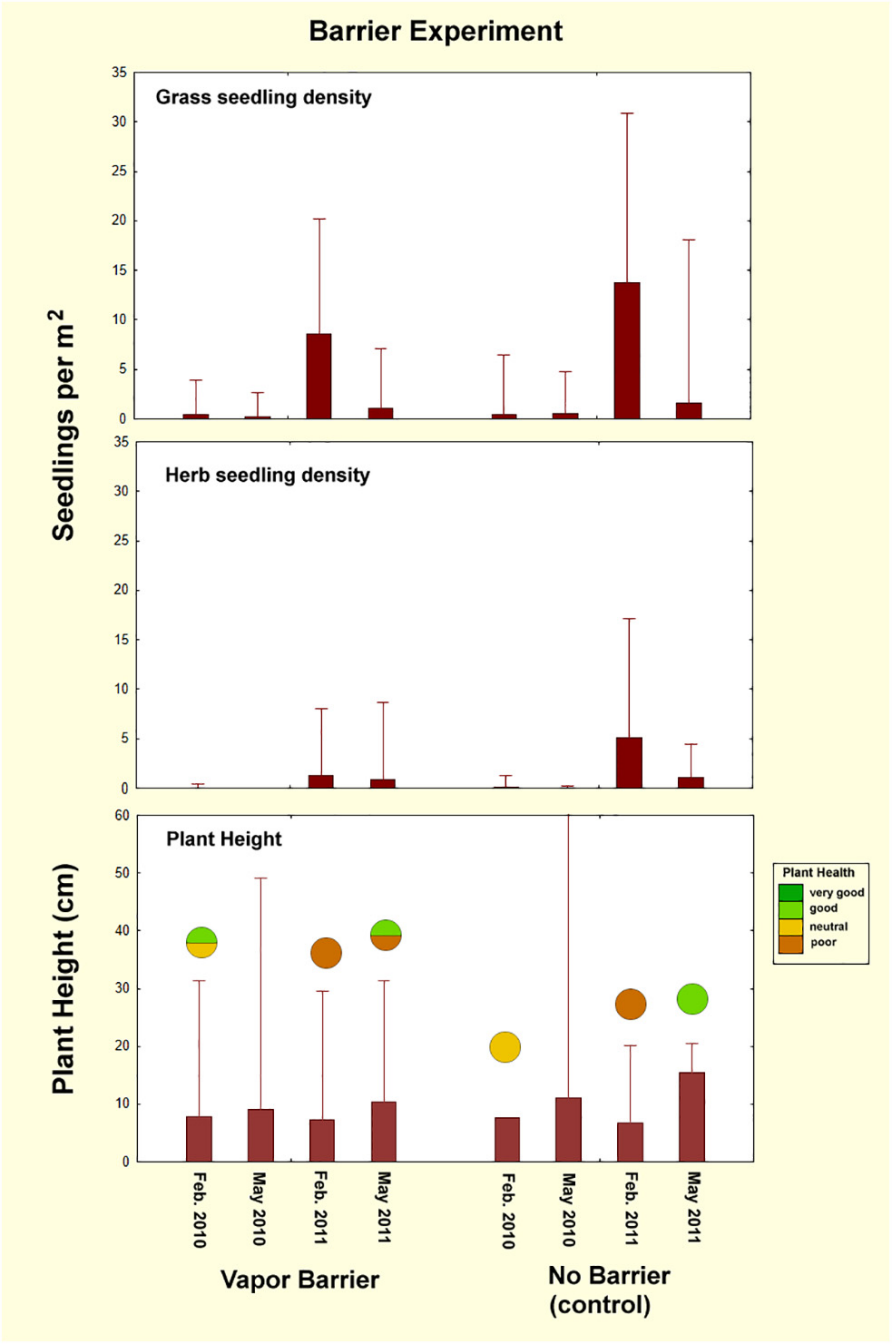
The results of the vapor barrier experiment on plant seedling density and growth. Placing a vapor barrier at a depth of 30 cm did not affect seedling density in comparison to a barrier-less control, nor did it affect plant growth. The fraction of fairy circles with the general plant health indicated by the color is shown by the circles in the lowest panel. In some cases, data were missing for one replicate, and no plant health data were available for May 2010.

Although both grass and herb seedling density changed significantly across check dates (ANOVA: grass, F_1,3_ = 108; herbs, F_1,3_ = 22; both p< 0.001) (Table 2), the fairy circles with the buried barrier did not differ from the controls in seedling density (grass, F_1,3_ = 94; p>0.06; herbs, F_1,3_ = 9.49; p>0.9) (Fig 6). The barrier treatment also had no effect on plant height, relative to the controls (p>0.8), although plant height differed greatly among check dates (F_1,3_ = 1425; p<0.001). In the 4 fairy circles of this experiment, general plant health ranged from poor to good, and was more related to check date than to treatment (Fig 6, bottom panel).

**Table 2:** Barrier Experiment, repeated measures analysis of variance. Significant effects are shown in bold.

		SS	d.f.	MS	F	p
Grass seedlings per m^2^	Intercept	**143.7**	**1**	**143.7**	**1045**	**0.020**
	Treatments	12.9	1	12.9	94	0.065
	Error	0.1	1	0.1		
	Check date	**240.2**	**3**	**80.1**	**108**	**0.001**
	Date by Treatments	17.9	3	6.0	8	0.060
	Error	2.2	3	0.7		
Herb seedlings per m^2^	Intercept	**17.85**	**1**	**17.85**	**42.03**	**0.023**
	Treatments	4.03	1	4.03	9.49	0.091
	Error	0.85	2	0.42		
	Check date	**26.87**	**3**	**8.96**	**21.95**	**0.001**
	Date by Treatments	**10.45**	**3**	**3.48**	**8.54**	**0.014**
	Error	2.45	6	0.41		
Grass height, cm	Intercept	644.4	1	644.4	35	0.106
	Treatments	1.9	1	1.9	0	0.800
	Error	18.4	1	18.4		
	Check date	**57.0**	**2**	**28.5**	**1425**	**0.001**
	Date by Treatments	**14.1**	**2**	**7.0**	**352**	**0.003**
	Error	0.0	2	0.0		

**Fig 7:**
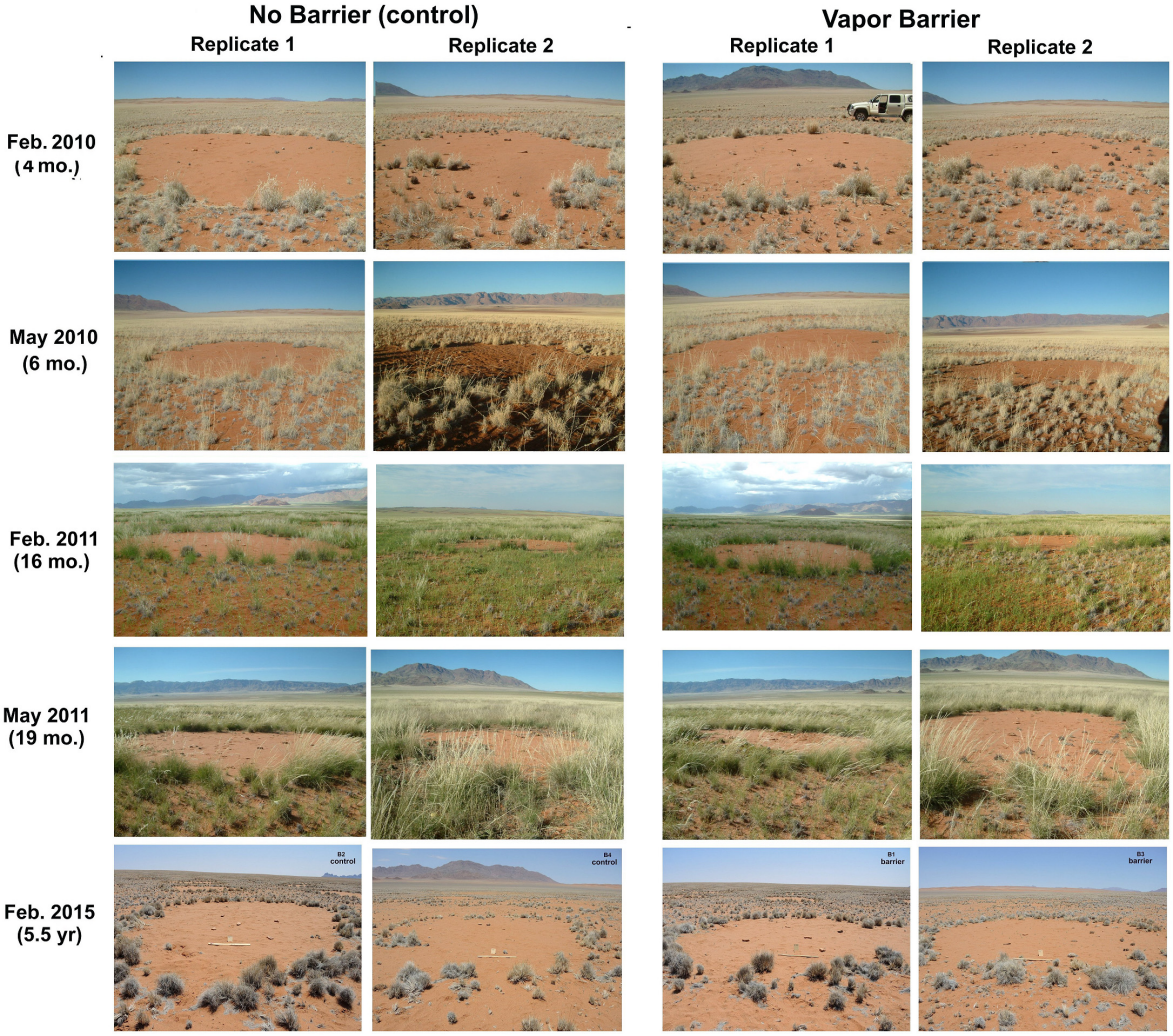
The appearance of the barrier experiment circles from Feb. 2010 until Feb. 2015. Presence of the vapor barrier 30 cm below the surface did not cause revegetation. There is no evidence that subterranean vapors or gasses are the cause of the bare circles.

Fig 7 shows the condition of the two replicates of the barrier experiment from February 2010 to February 2015, including the high rainfall period of early 2011. At no time did the vegetation in the barrier treatments differ visibly from that in the control. Even in the May 2011 sample in which grass growth was lush throughout the NRNR, no grasses populated either the barrier treatments or the control, nor had any growth occurred five years later in 2015 at which time there were no differences in the appearance of the circles with and without barriers.

### Micronutrients experiment

**Fig 8:**
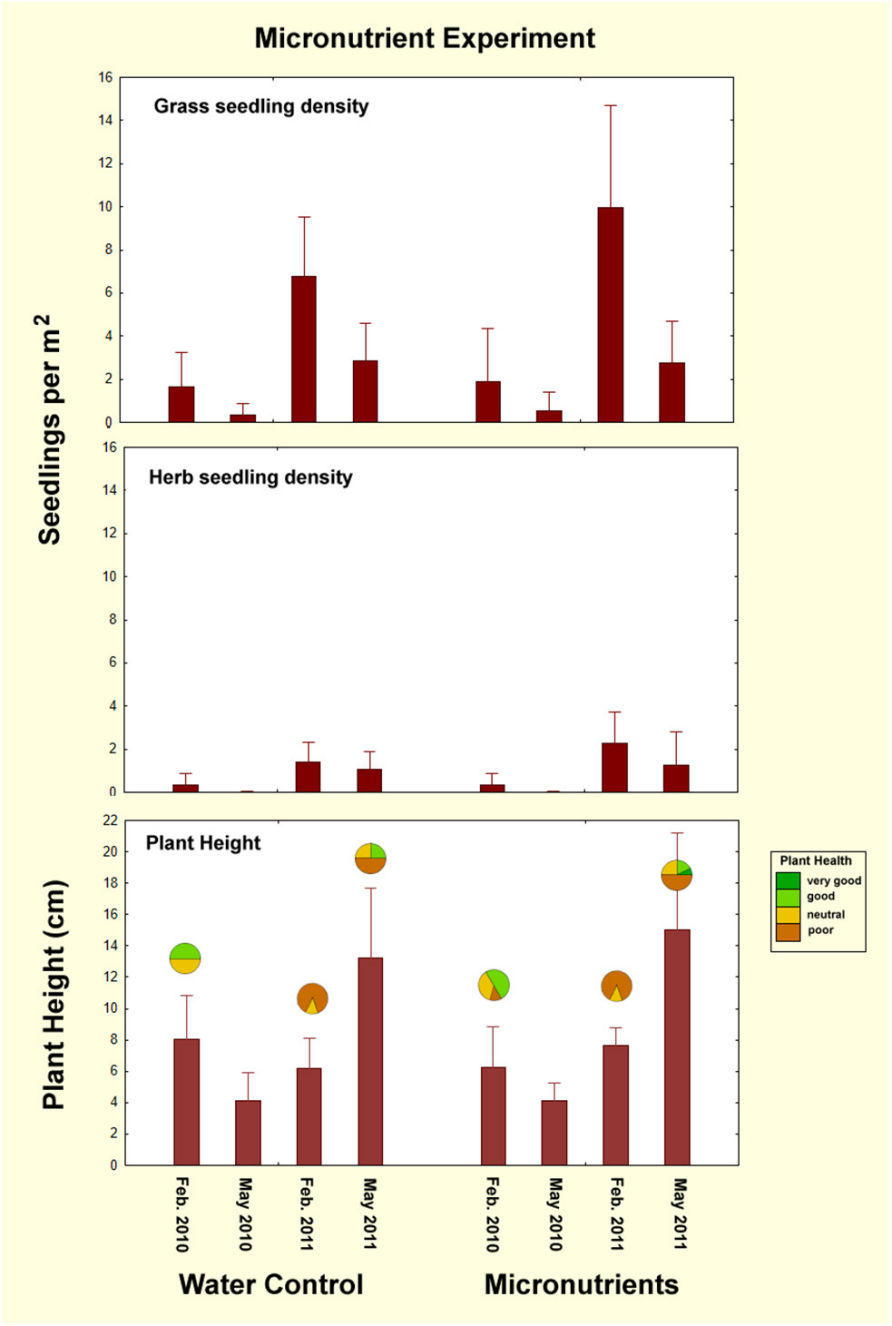
Seedling density and height in the micronutrient experiment. Addition of micronutrients had no effect (Table 3) on the density of either grass or herb seedlings, nor on plant growth, although all changed greatly over time. The addition of micronutrients had no significant effect on plant height, although height differed significantly by date. The fraction of fairy circles with the general plant health indicated by the color is shown by the circles in the lowest panel. In some cases, data were missing for one replicate.

**Fig 9:**
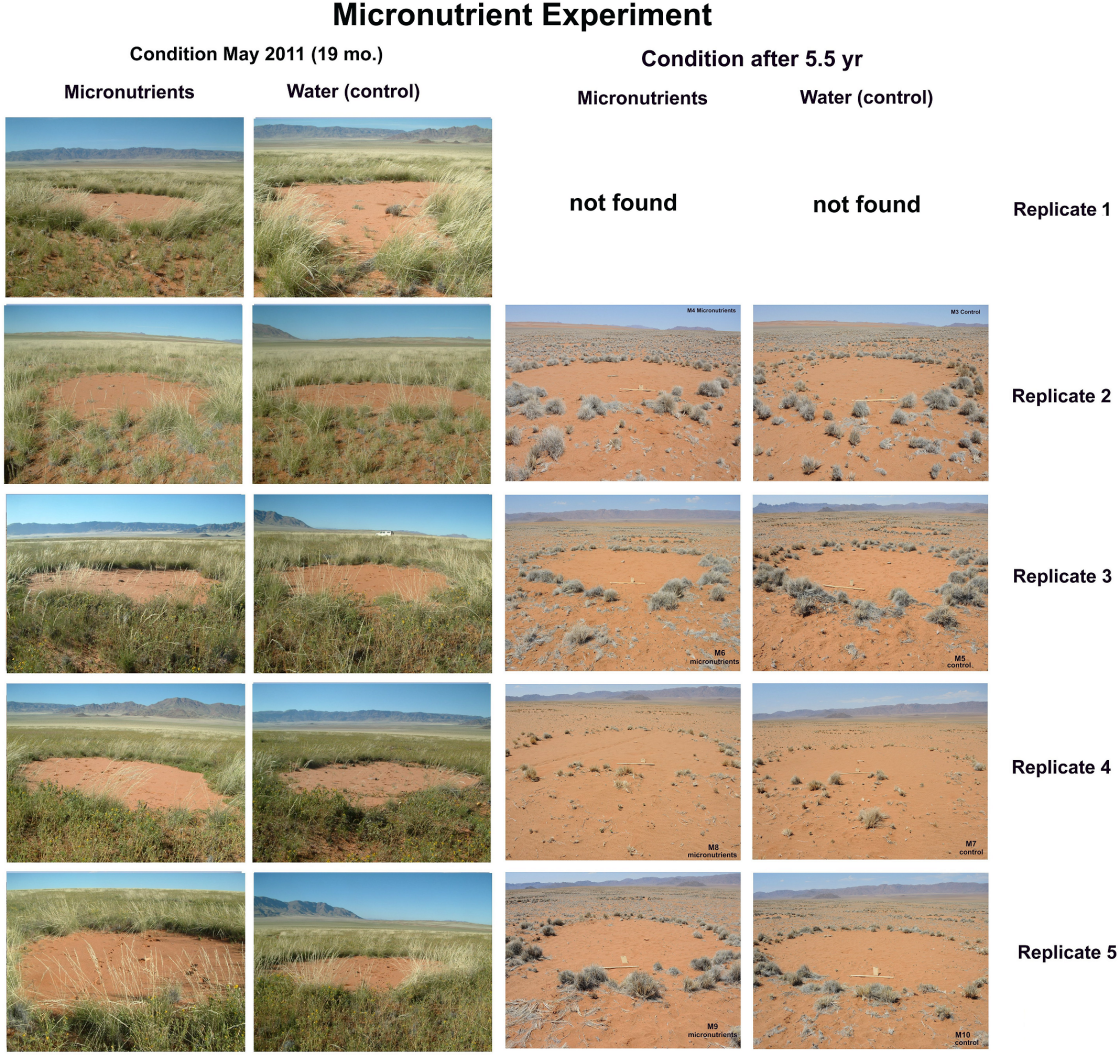
The condition of the micronutrient and control-treated fairy circles, replicates 1–5 in the high growth period in May 2011 and 5.5 yr after establishment. Replicate 1 was not found in 2015. Treatment with micronutrients did not stimulate or allow vegetation growth within the circles, suggesting that micronutrient-depletion is not a cause of fairy circle bareness. Only 5 of the 9 replicate pairs and two of the time samples are shown here. The pairs not shown were all very similar to these.

Addition of micronutrients to fairy circles did not significantly change either seedling density or plant growth (ANOVA: grass, F_1,3_ = 0.74; p>0.4; herbs, F_1,3_ = 0.22; p>0.4; n.s.) (Fig 8), although all measures differed significantly across check dates (grass, F_1,3_ = 24.7; herbs, F_1,3_ = 9.28; both p< 0.0001) (Table 3). Height growth between February and May of 2011 was especially apparent (F_1,3_ = 9.9; p<0.001), but such growth was similar for treatments and controls (p> 0.9). In the images of the nine pairs of treated circles, no visible differences in the growth of vegetation between treatment and controls are apparent, even in the high growth year of 2011 (Fig 9). Of the 18 circles in the experiment, plant health varied from poor to good but appeared unrelated to treatment (Fig 8, bottom panel). Five years after supply of micro-nutrients, there were no differences between treated and control circles.

**Table 3:** Micronutrients experiment, repeated measures analysis of variance. Significant effects are shown in bold.

		SS	d.f.	MS	F	p
**Grass seedlings per m2**	Intercept	**776.7**	**1**	**776.7**	**53.95**	**0.000002**
	Treatments	10.7	1	10.7	0.74	0.4
	Error	215.9	15	14.4		
	Check date	**612.8**	**3**	**204.3**	**24.66**	**0.000000**
	Date by Treatments	32.8	3	10.9	1.32	0.279138
	Error	372.8	45	8.3		
**Herb seedlings per m2**	Intercept	**35.39**	**1**	**35.39**	**29.18**	**0.000**
	Treatments	0.26	1	0.26	0.22	0.6
	Error	15.77	13	1.21		
	Check date	**27.32**	**3**	**9.11**	**9.28**	**0.000**
	Date by Treatments	2.85	3	0.95	0.97	0.417
	Error	38.29	39	0.98		
**Grass height, cm**	Intercept	**4033**	**1**	**4033**	**274.4**	**0.000**
	Treatments	0	1	0	0.0	0.929
	Error	206	14	15		
	Check date	**423**	**2**	**212**	**9.9**	**0.001**
	Date by Treatments	21	2	10	0.5	0.619
	Error	596	28	21		

### Soil transfer experiment

**Fig 10:**
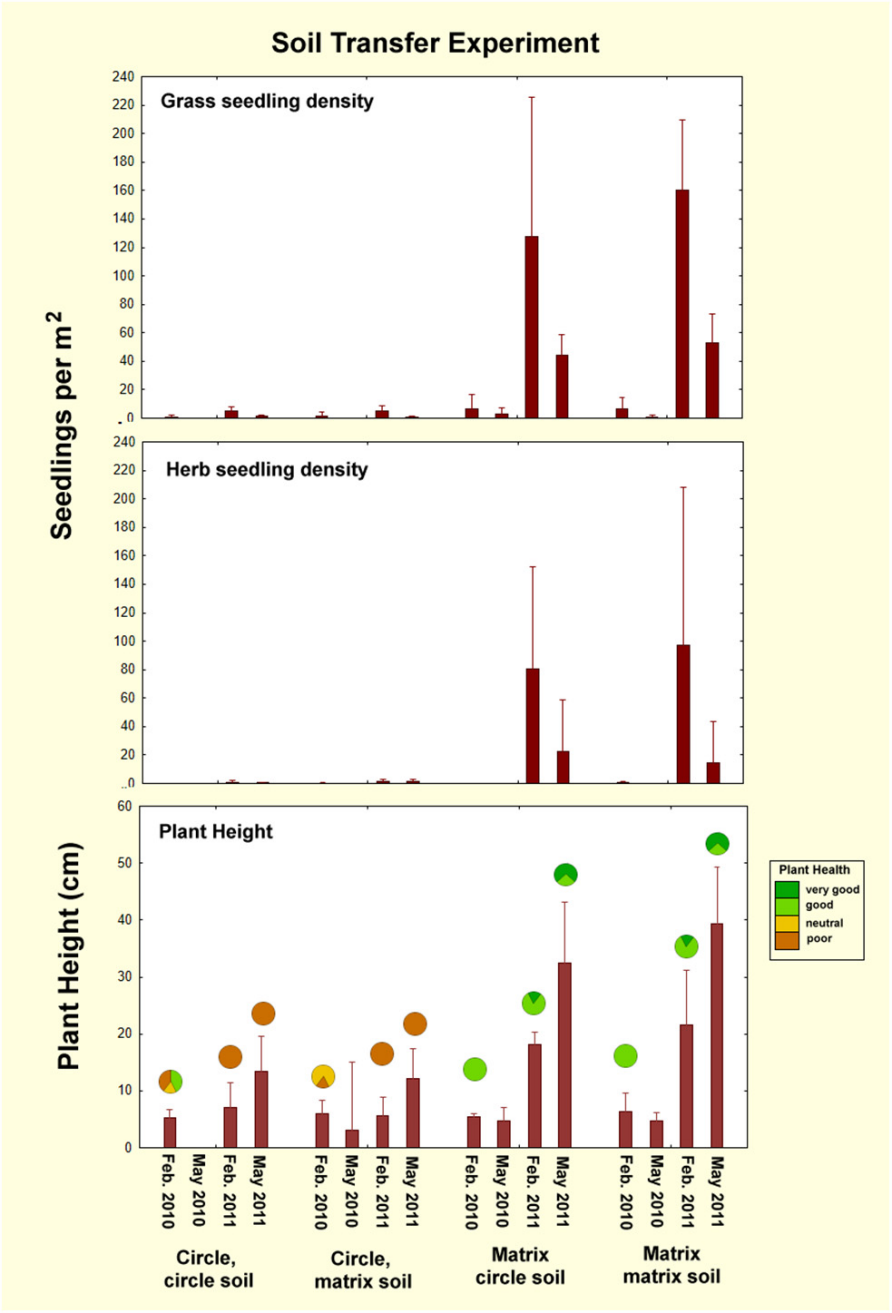
Results of the soil transfer experiment. Transfer of circle soil to the matrix did not change seedling density or growth from the reference circle-to-circle treatment (reference condition 1), nor did transfer of matrix soil to circles change seedling density or growth from the matrix-to-matrix treatment (reference condition 2). There is thus no evidence that circle soil inhibits plant seedling density or growth. The fraction of fairy circles with the general plant health indicated by the color is shown by the circles in the lowest panel. In some cases, data were missing for one replicate.

There was no evidence that circle soil reduced seedling density or growth (p> 0.9; Fig 10; Table 4). Although seedling density was significantly different across treatments (ANOVA: grass, F_1,3_ = 26.0; p<0.00005; herbs, F_1,3_ = 12.0; p<0.0005), this resulted from a lack of change from the initial condition, as there was very low seedling density in the natural circles, and 5 to 50 times as much in the artificially-created matrix circles. Transfer of natural circle soil to the artificial matrix circles did not change seedling density or growth from that of the reference matrix-to-matrix treatment (reference condition 2), nor did transfer of matrix soil to circles change seedling density or growth from that of the circle-to-circle treatment (reference condition 1). As in all other experiments, all measures differed significantly across check dates (grass, F_1,3_ = 46.9; herbs, F_1,3_ = 10.7; both p< 0.0001) (Table 4). Height growth between February and May of 2011 was especially apparent (F_1,3_ = 102; p<0.00001), but such growth was similar for treatments and controls. In almost all cases, plants were assessed as having poor to neutral health in the natural fairy circles of either treatment, and good to very good health in the artificial matrix circles. Only 2 of 5 circle-to-circle treatments in February 2010 enjoyed good health. On the other hand, plants in the former matrix circles all enjoyed good plant health in all years, without exception.

**Table 4:** Soil transfer experiment, repeated measures analysis of variance. Significant effects are shown in bold.

		SS	d.f.	MS	F	p
**Grass seedlings per m2**	Intercept	**54125**	**1**	**54125**	**87.85**	**0.000000**
	Treatments	**48028**	**3**	**16009**	**25.98**	**0.000002**
	Error	9858	16	616		
	Check date	**69278**	**3**	**23093**	**46.93**	**0.000000**
	Date by Treatments	**63241**	**9**	**7027**	**14.28**	**0.000000**
	Error	23618	48	492		
**Herb seedlings per m2**	Intercept	**16549**	**1**	**16549**	**37.24**	**0.0000**
	Treatments	**15989**	**3**	**5330**	**11.99**	**0.0005**
	Error	5777	13	444		
	Check date	**29373**	**3**	**9791**	**10.72**	**0.0000**
	Date by Treatments	**30735**	**9**	**3415**	**3.74**	**0.0018**
	Error	35635	39	914		
**Grass height, cm**	Intercept	**12473**	**1**	**12473**	**366.2**	**0.0000**
	Treatments	**2374**	**3**	**791**	**23.23**	**0.0000**
	Error	545	16	34		
	Check date	**3530**	**2**	**1765**	**102.2**	**0.0000**
	Date by Treatments	**1368**	**6**	**228**	**13.2**	**0.0000**
	Error	553	32	17		

**Fig 11:**
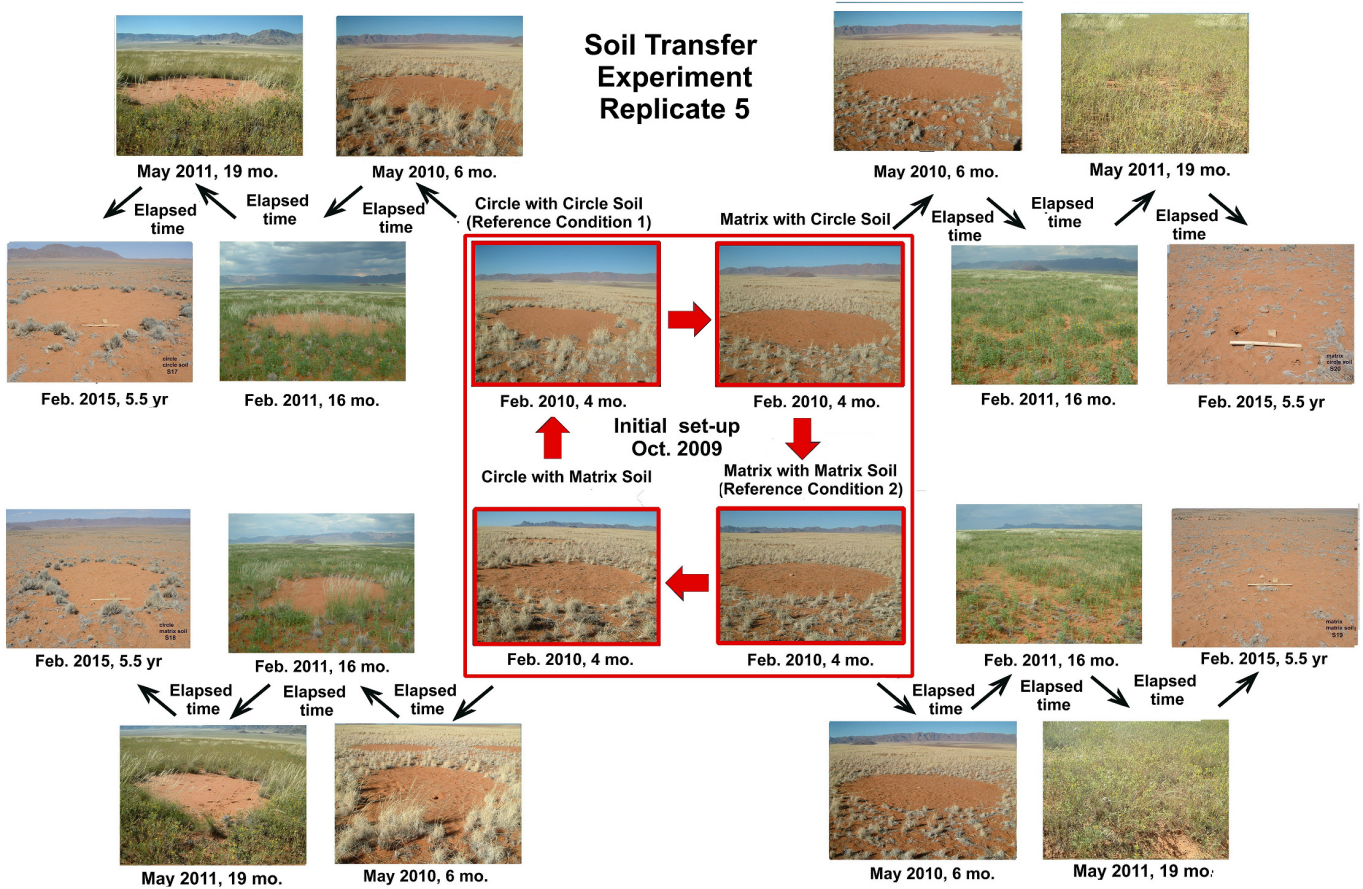
The results of the soil transfer experiment for replicate 5 over the 5 year duration of monitoring. Transfer of circle soil to matrix did not inhibit vegetation, nor did transfer of matrix soil to circles stimulate it. There was thus no evidence that circle soil inhibits vegetation. The four replicates not shown were very similar in appearance.

The condition of the five replicates of this experiment was similar at each of the five revisits, so images from only a single replicate are shown in Fig 11. In no replicate did the transfer of circle soil to matrix visibly inhibit matrix vegetation, nor did transfer of matrix soil to circles dis-inhibit or stimulate circle vegetation. In all cases, the matrix revegetated normally, especially during the high growth in May 2011, whereas the circles remained bare. There was thus no evidence that circle soil inhibited plant growth.

### Neighborhood experiment

**Fig 12:**
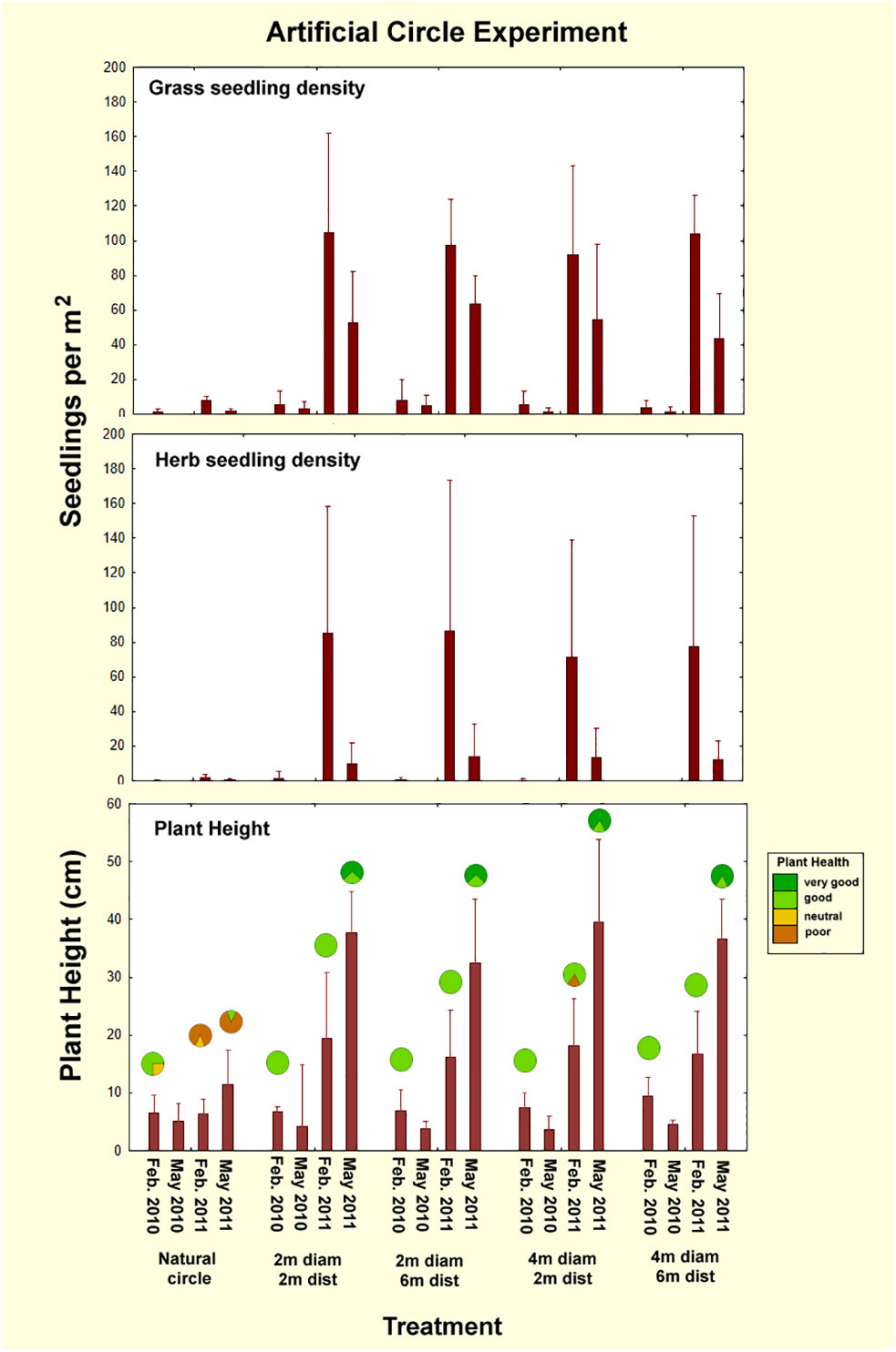
Plant seedling density and growth in the neighborhood experiment from February 2010 to May 2011. With adequate rain in 2011, all artificial circles created in the matrix revegetated. Moreover, plant health was better in the matrix circles, almost without exception. The fraction of fairy circles with the general plant health indicated by the color is shown by the circles in the lowest panel. In some cases, data were missing for one replicate. No plant health data were available for May 2010.

Artificial circles cleared in the matrix reverted to matrix regardless of their size or distance from the central natural fairy circle. Seedling density was significantly different across treatments (Table 5; ANOVA: grass, F_1,3_ = 6.15; p<0.003; herbs, F_1,3_ = 1.5; p>0.2; n.s.) because there was little germination in the natural circles, compared to the abundant germination in the artificially-created matrix circles. This reversion occurred most dramatically after the abundant rains in 2011, and can be seen in the high seedling density and growth in the artificial circles (Fig 12). Although seedling density changed across check dates (grass, F_1,3_ = 78.1; p<0.00001; herbs, F_1,3_ = 18.1; p<0.00001), there was no difference among artificial circles for any given date. As with all other experiments, plant height changed significantly across check dates (F_1,3_ = 42.9; p<0.0000), but this change was similar for all treatments (Fig 12). Just as there was no effect on the artificial circles by the natural circle, there was no effect by the artificial circles on the natural, as indicated by the lack of change in perimeter grass height from control natural circles, and in the lack of significant change in the 5 natural circle diameters as measured on Google Earth images taken 2009, 2010, 2011, 2013 and 2015.

**Table 5:** Artificial circle experiment, repeated measures analysis of variance. Significant effects are shown in bold.

		SS	d.f.	MS	F	p
**Grass seedlings per m2**	Intercept	**88912**	**1**	**88912**	**146.8**	**0.000000**
	Treatments	**14883**	**4**	**3720**	**6.15**	**0.003**
	Error	9687	16	605		
	Check date	**88157**	**3**	**29386**	**78.05**	**0.000000**
	Date by Treatments	**15957**	**12**	**1330**	**3.53**	**0.0009**
	Error	18071	48	377		
**Herb seedlings per m2**	Intercept	**24631**	**1**	**24631**	**31.67**	**0.0000**
	Treatments	4670	4	1167	1.501	0.2488
	Error	12445	16	778		
	Check date	**52137**	**3**	**17379**	**18.05**	**0.0000**
	Date by Treatments	10227	12	852	0.885	0.5675
	Error	46226	48	963		
**Grass height, cm**	Intercept	**7633**	**1**	**7633**	**220.3**	**0.000**
	Treatments	551	4	138	4.0	0.065
	Error	208	6	35		
	Check date	**4867**	**3**	**1622**	**42.9**	**0.000**
	Date by Treatments	878	12	73	1.9	0.100
	Error	680	18	38		

**Fig 13:**
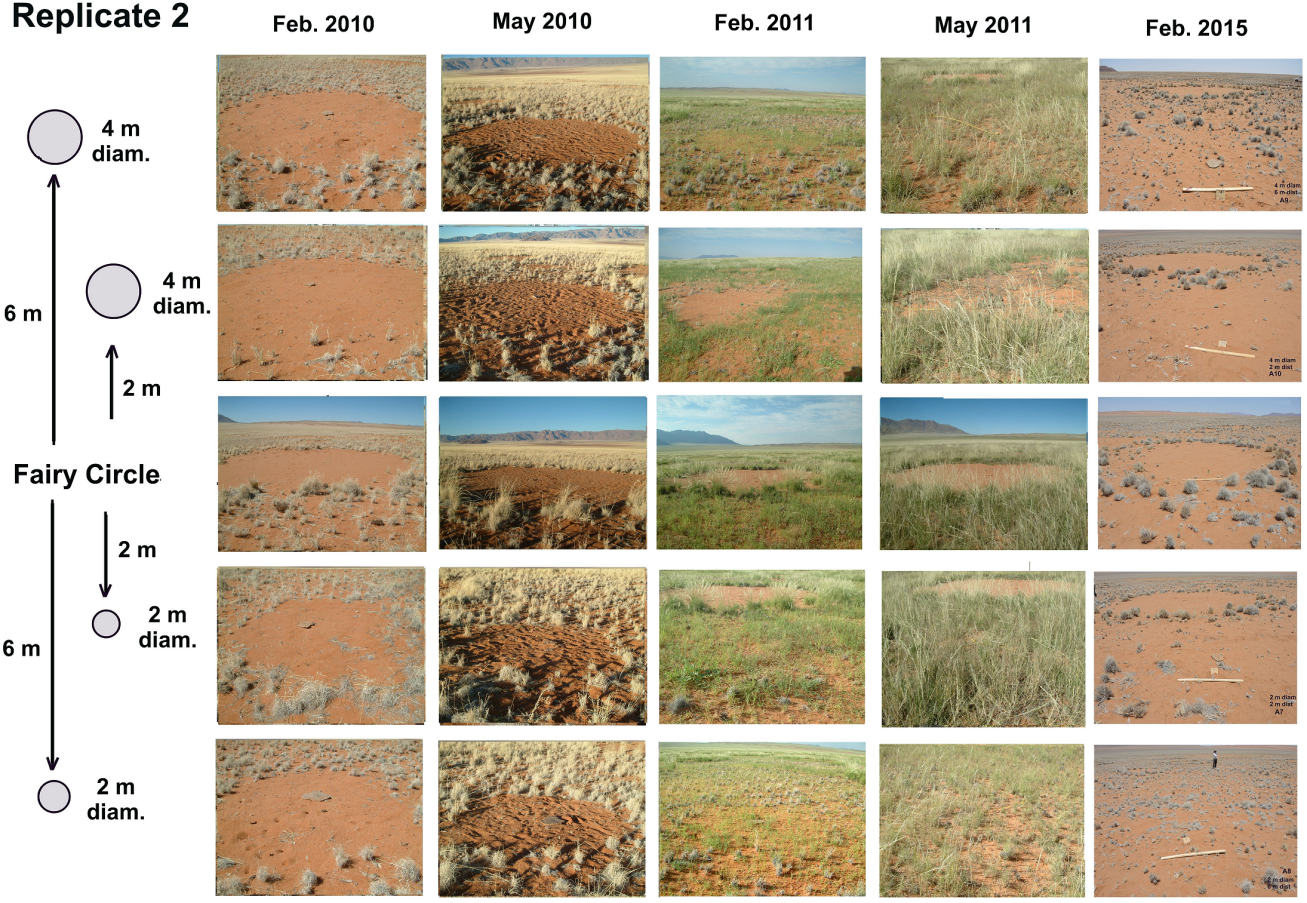
Replicate 2 of the neighborhood experiment showing condition from Feb. 2010 to Feb. 2015. The artificial circles of 2 or 4 m diameter and 2 to 4 m distant all revegetated during the wet May of 2011. The artificial circles did not affect the central, natural fairy circle. All five replicates were similar. This experiment did not suggest neighborhood effects on this scale. No images of February 2011 were available.

Plant health in the natural fairy circles followed the general trends in that the majority exhibited poor to neutral health in all three checks, while 80 to 100% of the artificially-created matrix circles enjoyed good to very good health as they revegetated (Fig 12, bottom panel). In contrast, the artificial circles, no matter what their diameter or distance from the natural fairy circle, filled in during the wet May of 2011 so that no trace of them remained in Feb. 2015 (Fig 13). There was also no apparent influence of these artificial circles on the central, natural fairy circle. Thus, removal of matrix vegetation in the vicinity of fairy circles created no obvious neighborhood effects on this scale. The emergent fairy circle patterns thus seem resilient to perturbation on this small scale, but the experiment does not address the larger landscape scale.

### Biomass

ANOVA of the biomass index (seedling density x height) did not change any of the conclusions presented above on the basis of seedling density and growth.

## Discussion

Given the consistent spatial regularity of fairy circle dispersions over their geographic range, their predictable size on the basis of latitude, climate, soil type, local density/spacing, and their temporal variability [2, 18, 19, 28] it is clear that explanations that do not address all of these attributes cannot be considered correct and/or complete. The capacity to do so should thus be the accepted standard applied to all hypotheses. Comparison of competition model outcomes to satellite or aerial images has shown consistent agreement with the known fairy circle patterns in both space and time [18, 19, 28, 29], but to date, similar consistency of other hypotheses with these multiple fairy circle attributes has not been demonstrated, or in many cases, not attempted.

Causation by toxic gases or vapors emanating from subterranean hydrocarbon seeps or termite nests [3, 6, 7, 10] clearly falls short on this account, as was clearly explained by Getzin et al. [28], since the hypothesis offers no explanation of the regularity, regional occurrence and size of the circles, nor their dynamic nature. Naude et al. (unpublished lecture pdf) draw on a study of "pockmarks" caused by gas seeps [31] as having the capacity to explain the overdispersion of fairy circles, but the pockmarks are on the deep sea bed of the Nile Delta, and unlike fairy circles, are not spatially homogeneous. In our experiment, seedling density was similar in barrier and control circles. Thus, if toxic gases or vapors were the cause, the subterranean vapor barrier should have resulted in at least some plant survival and growth above the barrier, but not the control. However, there was no difference between them. Although the barrier is unlikely to be completely impermeable to gases and organic molecules, it would be expected to reduce the vertical gas flux and displace the flux to the edges of the barrier, resulting in partial revegetation over the center of the barrier. The lack of any differences between control and barrier treatments, however, eliminates such vapors as a possible cause of fairy circles.

Although some termites harvest grass [4, 16, 32, 33], and some social insects are capable of establishing exclusive territories, any mechanism invoking termites must also show that (1) colonies are distinct entities on a scale similar to the temporal and spatial scale of fairy circles, and that these entities reject each other's workers, or are limited to their own territory by some other mechanism; (2) the interactions among colonies causes them, or at least their territories, to be regularly dispersed, so that the hypothesized sources of toxic vapors are also regularly dispersed; (3) the feeding or harvesting or other actions of the termites result in grass death, or prevent grass reproduction, or do something else (vapors?) that results in a circular bare area; (4) colonies could (in theory) produce sufficient quantities of the putative toxin, given its toxicity; (5) the "nests" that are detected represent (or not) a distinct colony. Seen in this light, it is unlikely that termites or their products cause fairy circles, either through direct action or toxic emissions [4,10,12,30,33]. While termites can certainly be expected to feed on the dying grasses of any forming fairy circle, the evidence that termites *cause* fairy circles currently remains weak to absent.

Our soil transfer experiment suggested that fairy circle soil did not reduce plant seedling density and growth under field conditions, and that matrix soil did not stimulate it. This is consistent with the findings of Jankowitz et al. [7] who found that location, rather than soil source, determined plant health. Previous comparisons of growth on fairy circle compared to matrix soils in glass houses used commercially available seeds of grass, alfalfa or wheat [1–3, 13] rather than the native *Stipagrostis* spp., raising the issue of relevance to field conditions. At most, these studies indicate that under well-watered conditions, growth of phytometers is impaired by fairy circle soils. In the field, however, other factors likely dominate inherent differences in soils. We suggest that this factor is water limitation, the availability of which is partially determined by location of the plants in either a fairy circle or in the matrix. It could be argued that in our transfer experiment, insufficient soil was transferred to sustain an effect, but our seedling density and growth measurements were quantitative over the first years and thus capable of detecting small changes in plant growth. A general way to interpret this experiment is that the vegetation is absent because some non-transferrable, cryptic factor determines where plants can grow and where they cannot. This factor is a characteristic of the landscape itself.

Poorer growth of phytometers on fairy circle soils compared to matrix soils in green house studies [1, 2] may have resulted from micronutrient differences. Micronutrient deficiencies have been reported to restrict plant growth in Australia [34] and other areas with infertile soils, suggesting that some redistribution mechanism might create spots so locally depleted in micronutrients that they could no longer support plant growth. However, supplementation of fairy circles with a mixture of micronutrients did not stimulate growth over controls, and thus did not support this hypothesis. Moll [3] reported nutrient analyses of fairy circle and matrix soil at Kamberg, but provided neither statistical tests nor conclusions. Our reanalysis of his data, including correcting for difference in loss on ignition, revealed no significant (p<0.05, Student’s t test, n = 5) differences across macronutrients (Mg, Ca, K) or micronutrients (Mn, P, Fe, Zn Cu, Ni, Co). It is thus unlikely that micronutrient deficiencies explain fairy circles.

The strongest hypothesis currently under consideration is that positive and negative feedbacks resulting from plant competition in this arid land bring about the self-organization of this grassland into a vegetated matrix with bare gaps by reducing resources in these gaps below what can sustain vegetation. Several models that apply such mechanisms are capable of simulating patterned vegetation, including gaps [18–29, 35, 36]. Once these gaps are formed, the greater abundance of water allows the more water-requiring and taller *S*. *ciliata* to colonize the circle perimeter. Subsequently, the continued withdrawal of water (and possibly nutrients) by the combined effect of the matrix and perimeter grasses maintains the bareness of the fairy circles. Removal of vegetation in close proximity (i.e. 2 m) to a natural fairy circle might have been expected to increase the growth of peripheral grass on the fairy circle, but there was no evidence of this. Clearing an area of vegetation in the matrix might also have been expected to provide the stimulus for the initiation of a new fairy circle, but vegetation of these cleared areas recovered rapidly. Thus, whatever interactions create fairy circles, they do not occur on the scale of 2 to 10 m, and require more than mere localized removal of existing vegetation or moving of soil. We conclude that the disturbances in the landscape patterns of water availability introduced by clearing were insufficient to alter peripheral vegetation on existing fairy circles or to generate new fairy circles. Clearing of 2–4 m diameter patches perhaps represents a rather minor perturbation of water availability. Recent modeling and comparison with satellite images has shown that fairy circles are more likely to form where neighborhood distances and water stress are greater (S. Getzin, pers. comm.), but in 2009 we had no a priori knowledge of where those areas might be, and in any case, we established these experiments long before these models were published.

## Supporting Information

S1 Table Description, location, condition and designation of the experimental circles. (DOCX)

S1 Data Monitoring data for 2010 and 2011, showing treatments, conditions, seedling density, plant height and other variables. (XLS)
