# Supplementary material for: Experiments Testing the Causes of Namibian Fairy Circles
Source: PLoS One. 2015 Oct 28;10(10):e0140099. doi: 10.1371/journal.pone.0140099 (PMC4625006; doi:10.1371/journal.pone.0140099)
Supplement: S1 Table — (DOCX) [file pone.0140099.s001.docx]

S1 Table. Description, location, condition and designation of the experimental circles.

| \| Experiment \| \| --- \| | \| Expmtl. designation \| \| --- \| | \| Treatments \| \| --- \| | \| Diameter (m) \| \| --- \| | \| Initial bareness \| \| --- \| | \| Concavity, cm \| \| --- \| | \| No. tall grass clumps in perimeter \| \| --- \| | \| Latitude \| \| --- \| | \| Longitude \| \| --- \| |
| --- | --- | --- | --- | --- | --- | --- | --- | --- | --- | --- | --- | --- | --- | --- | --- | --- | --- |
| artificial circles | A1 | Natural circle | 9 | inner ring, live | 0 | 27 | -24.98092 | 15.96514 |
| artificial circles | A2 | 2m diam, 2m dist | 2 | matrix | 0 | 0 | -24.98086 | 15.96517 |
| artificial circles | A3 | 4m diam, 6m dist | 4 | matrix | 0 | 0 | -24.98086 | 15.96503 |
| artificial circles | A4 | 2m diam, 6m dist | 2 | matrix | 0 | 0 | -24.98100 | 15.96511 |
| artificial circles | A5 | 4m diam, 2m dist | 4 | matrix | 0 | 0 | -24.98095 | 15.96522 |
| artificial circles | A6 | Natural circle | 10 | completely bare | 3 | 40 | -24.98189 | 15.96225 |
| artificial circles | A7 | 2m diam, 2m dist | 2 | matrix | 0 | 0 | -24.98182 | 15.96222 |
| artificial circles | A8 | 2m diam, 6m dist | 2 | matrix | 0 | 0 | -24.98183 | 15.96235 |
| artificial circles | A9 | 4m diam, 6m dist | 4 | matrix | 0 | 0 | -24.98196 | 15.96236 |
| artificial circles | A10 | 4m diam, 2m dist | 4 | matrix | 0 | 0 | -24.98186 | 15.96217 |
| artificial circles | A11 | Natural circle | 9 | completely bare | 11 | 18 | -24.97699 | 15.94960 |
| artificial circles | A12 | 2m diam, 2m dist | 2 | matrix | 0 | 0 | -24.97693 | 15.94958 |
| artificial circles | A13 | 2m diam, 6m dist | 2 | matrix | 0 | 0 | -24.97699 | 15.94970 |
| artificial circles | A14 | 4m diam, 2m dist | 4 | matrix | 0 | 0 | -24.97703 | 15.94964 |
| artificial circles | A15 | 4m diam, 6m dist | 4 | matrix | 0 | 0 | -24.97708 | 15.94955 |
| artificial circles | A16 | Natural circle | 7 | completely bare | 9 | 18 | -24.98124 | 15.95859 |
| artificial circles | A17 | 2m diam, 2m dist | 2 | matrix | 0 | 0 | -24.98119 | 15.95862 |
| artificial circles | A18 | 4m diam, 2m dist | 4 | matrix | 0 | 0 | -24.98128 | 15.95866 |
| artificial circles | A19 | 2m diam, 6m dist | 2 | matrix | 0 | 0 | -24.98134 | 15.95860 |
| artificial circles | A20 | 4m diam, 6m dist | 4 | matrix | 0 | 0 | -24.98116 | 15.95852 |
| artificial circles | A21 | Natural circle | 9 | completely bare | 13 | 23 | -24.97919 | 15.95382 |
| artificial circles | A22 | 2m diam, 2m dist | 2 | matrix | 0 | 0 | -24.97911 | 15.95383 |
| artificial circles | A23 | 2m diam, 6m dist | 2 | matrix | 0 | 0 | -24.97915 | 15.95391 |
| artificial circles | A24 | 4m diam, 2m dist | 6 | matrix | 0 | 0 | -24.97924 | 15.95386 |
| artificial circles | A25 | 4m diam, 6m dist | 4 | matrix | 0 | 0 | -24.97922 | 15.95373 |
| barrier | B1 | barrier |  | completely bare |  |  | -24.98095 | 15.96564 |
| barrier | B2 | no barrier |  | completely bare |  |  | -24.98099 | 15.96549 |
| barrier | B3 | barrier |  | completely bare |  |  | -24.98176 | 15.96208 |
| barrier | B4 | no barrier |  | completely bare |  |  | -24.98195 | 15.96203 |
| micronutrients | M1 | water control | 6 | completely bare | 3 | 30 | -24.98064 | 15.96519 |
| micronutrients | M2 | micronutrients | 8 | completely bare | 2 | 27 | -24.98048 | 15.96525 |
| micronutrients | M3 | micronutrients | 8 | completely bare | 5 | 10 | -24.98169 | 15.96186 |
| micronutrients | M4 | water control | 7 | completely bare | 0 | 18 | -24.98182 | 15.96176 |
| micronutrients | M5 | water control | 8 | completely bare | 16 | 31 | -24.97674 | 15.94955 |
| micronutrients | M6 | micronutrients | 8 | completely bare | 13 | 36 | -24.97678 | 15.94970 |
| micronutrients | M7 | water control | 8 | completely bare | 8 | 8 | -24.98092 | 15.95836 |
| micronutrients | M8 | micronutrients | 7 | completely bare | 7 | 16 | -24.98104 | 15.95842 |
| micronutrients | M9 | micronutrients | 6 | completely bare | 12 | 17 | -24.97934 | 15.95368 |
| micronutrients | M10 | water control | 8 | completely bare | 19 | 25 | -24.97933 | 15.95392 |
| micronutrients | M11 | water control |  | completely bare |  |  |  |  |
| micronutrients | M12 | micronutrients |  | completely bare |  |  |  |  |
| micronutrients | M13 | water control |  | completely bare |  |  |  |  |
| micronutrients | M14 | micronutrients |  | completely bare |  |  |  |  |
| micronutrients | M15 | water control |  | completely bare |  |  |  |  |
| micronutrients | M16 | micronutrients |  | completely bare |  |  |  |  |
| micronutrients | M17 | water control |  | completely bare |  |  |  |  |
| micronutrients | M18 | micronutrients |  | completely bare |  |  |  |  |
| soil transfer | S1 | circle, circle soil | 8 | completely bare | 6 | 19 | -24.98064 | 15.96499 |
| soil transfer | S2 | circle, matrix soil | 7 | completely bare | 0 | 14 | -24.98044 | 15.96498 |
| soil transfer | S3 | matrix, matrix soil | 6 | matrix | 0 | 0 | -24.98039 | 15.96485 |
| soil transfer | S4 | matrix, circle soil | 6 | matrix | 0 | 0 | -24.98053 | 15.96501 |
| soil transfer | S5 | circle, circle soil | 6 | completely bare | 4 | 35 | -24.98176 | 15.96225 |
| soil transfer | S6 | circle, matrix soil | 6 | completely bare | 4 | 18 | -24.98162 | 15.96218 |
| soil transfer | S7 | matrix, matrix soil | 6 | matrix | 0 | 0 | -24.98170 | 15.96217 |
| soil transfer | S8 | matrix, circle soil | 6 | matrix | 0 | 0 | -24.98163 | 15.96227 |
| soil transfer | S9 | circle, circle soil | 7 | completely bare | 18 | 21 | -24.97687 | 15.94936 |
| soil transfer | S10 | circle, matrix soil | 5 | completely bare | 9 | 8 | -24.97675 | 15.94937 |
| soil transfer | S11 | matrix, circle soil | 6 | matrix | 0 | 0 | -24.97681 | 15.94944 |
| soil transfer | S12 | matrix, matrix soil | 6 | matrix | 0 | 0 | -24.97680 | 15.94928 |
| soil transfer | S13 | circle, matrix soil | 8 | completely bare | 5 | 17 | -24.98144 | 15.95890 |
| soil transfer | S14 | circle, circle soil | 8 | completely bare | 6 | 17 | -24.98151 | 15.95903 |
| soil transfer | S15 | matrix, matrix soil | 6 | matrix | 0 | 0 | -24.98154 | 15.95891 |
| soil transfer | S16 | matrix, circle soil | 6 | matrix | 0 | 0 | -24.98159 | 15.95899 |
| soil transfer | S17 | circle, circle soil | 7 | completely bare | 17 | 20 | -24.97906 | 15.95406 |
| soil transfer | S18 | circle, matrix soil | 5 | completely bare | 8 | 5 | -24.97896 | 15.95400 |
| soil transfer | S19 | matrix, matrix soil | 6 | matrix | 0 | 0 | -24.97892 | 15.95409 |
| soil transfer | S20 | matrix, circle soil | 6 | matrix | 0 | 0 | -24.97899 | 15.95412 |
